# What’s a Biofilm?—How the Choice of the Biofilm Model Impacts the Protein Inventory of *Clostridioides difficile*

**DOI:** 10.3389/fmicb.2021.682111

**Published:** 2021-06-10

**Authors:** Madita Brauer, Christian Lassek, Christian Hinze, Juliane Hoyer, Dörte Becher, Dieter Jahn, Susanne Sievers, Katharina Riedel

**Affiliations:** ^1^Department for Microbial Physiology and Molecular Biology, Institute of Microbiology, University of Greifswald, Greifswald, Germany; ^2^Department for Microbial Proteomics, Institute of Microbiology, University of Greifswald, Greifswald, Germany; ^3^Braunschweig Integrated Centre of Systems Biology (BRICS), Institute of Microbiology, Technische Universität Braunschweig, Braunschweig, Germany

**Keywords:** biofilm, colony biofilm, aggregate biofilm, *Clostridioides difficile*, proteomics, cell surface antigens, RpoN signaling

## Abstract

The anaerobic pathogen *Clostridioides difficile* is perfectly equipped to survive and persist inside the mammalian intestine. When facing unfavorable conditions *C. difficile* is able to form highly resistant endospores. Likewise, biofilms are currently discussed as form of persistence. Here a comprehensive proteomics approach was applied to investigate the molecular processes of *C. difficile* strain 630Δ*erm* underlying biofilm formation. The comparison of the proteome from two different forms of biofilm-like growth, namely aggregate biofilms and colonies on agar plates, revealed major differences in the formation of cell surface proteins, as well as enzymes of its energy and stress metabolism. For instance, while the obtained data suggest that aggregate biofilm cells express both flagella, type IV pili and enzymes required for biosynthesis of cell-surface polysaccharides, the S-layer protein SlpA and most cell wall proteins (CWPs) encoded adjacent to SlpA were detected in significantly lower amounts in aggregate biofilm cells than in colony biofilms. Moreover, the obtained data suggested that aggregate biofilm cells are rather actively growing cells while colony biofilm cells most likely severely suffer from a lack of reductive equivalents what requires induction of the Wood-Ljungdahl pathway and *C. difficile’s* V-type ATPase to maintain cell homeostasis. In agreement with this, aggregate biofilm cells, in contrast to colony biofilm cells, neither induced toxin nor spore production. Finally, the data revealed that the sigma factor SigL/RpoN and its dependent regulators are noticeably induced in aggregate biofilms suggesting an important role of SigL/RpoN in aggregate biofilm formation.

## Introduction

In recent years, the anaerobic gastrointestinal pathogen *Clostridioides difficile* has established itself as one of the major causative agents of pseudomembranous colitis and toxic megacolon (Wiegand et al., 2012; Nasiri et al., 2018; Martínez-Meléndez et al., 2020; Doll et al., 2021). *C. difficile* does not only produce enterotoxins that damage the gastrointestinal epithelium (Fletcher et al., 2021) but also forms easily transmittable spores that significantly contribute to *C. difficile’s* efficient spreading in the environment, hospitals and elderly homes (McLure et al., 2019; Hernandez et al., 2020; Werner et al., 2020; Khader et al., 2021). If conditions are favorable, e.g., after antibiotic-induced dysbiosis when the reduced microbiome fails to convert primary bile acids into secondary bile acids, *C. difficile* spores are able to germinate to successively colonize the large intestine (Theriot et al., 2014; Pike and Theriot, 2020). Even though antibiosis mostly stops the acute infection, *C. difficile* spores as well as some vegetative cells survive in the intestine and can subsequently cause a relapse as soon as antibiotic concentrations are sufficiently low (Goulding et al., 2009; Chilton et al., 2018; Castro-Córdova et al., 2020; Feuerstadt et al., 2021; Normington et al., 2021). Initially, sporulation was assumed to be one of the major prerequisites for *C. difficile’s* persistence under clinical circumstances. However, recent publications failed to correlate sporulation efficiency of a certain strain with the corresponding virulence and persistence potential, i.e., highly infectious strains do not necessarily produce more spores than other strains (Sirard et al., 2011; Oka et al., 2012; Plaza-Garrido et al., 2015; Gómez et al., 2017). In conclusion, persistence does obviously not solely rely on sporulation but also on additional features of *C. difficile* (Smits, 2013). In this context, biofilm formation was proposed to be a major additional factor (Dawson et al., 2012; Ðapa et al., 2013). Surface-associated biofilms, consisting of multiple microorganisms embedded in a slimy extracellular matrix, represent a form of community lifestyle for many bacteria (Hansen et al., 2007; Oliveira et al., 2015; Ren et al., 2015). Many nosocomial infections caused by *Staphylococcus aureus*, *Streptococci* and numerous other pathogens rely on biofilms (Jamal et al., 2018). In this context, biofilm formation has been frequently linked to pro-longed infection and persistence (Burmølle et al., 2010; Jamal et al., 2018). An important feature of biofilms is their extracellular matrix, which mostly consists of extracellular DNA (eDNA), lipids, proteins and polysaccharides. The extracellular matrix provides protection against chemical and mechanical stress and confers resistance to therapeutics and the host immune system (Karygianni et al., 2020). Anaerobic bacteria, including *C. difficile* have also been found to produce biofilms *in vitro*, and biofilm-like structures have been observed on the intestinal mucosal surface of *C. difficile* infected mice (Donelli et al., 2012; Lawley et al., 2012; Soavelomandroso et al., 2017).

Initial biofilm studies using *C. difficile* revealed mechanisms of formation and involved components comparable to those determined for other bacteria (Dawson et al., 2012; Vuotto et al., 2018). For instance, the involvement of adhesion-mediating cell surface structures, such as flagella, pili and various adhesion molecules, has been reported (Reynolds et al., 2011; Ðapa et al., 2013; Pantaléon et al., 2015). Similarly, c-di-GMP signaling, quorum sensing, and regulators, such as Spo0A and CodY, are involved in coordination of the process of biofilm formation (Ðapa et al., 2013; Purcell et al., 2016; McKee et al., 2018b; Dubois et al., 2019). Knockout mutants of some genes encoding certain proteins commonly linked to biofilm formation, such as adhesion and cell signaling proteins, were found impaired in biofilm formation *in vitro* and revealed a reduced infection and persistence behavior in rodent models (Barketi-Klai et al., 2011; Dawson et al., 2012; Ðapa et al., 2013; Batah et al., 2017; McKee et al., 2018a; Slater et al., 2019). Corresponding cell-cell aggregates were observed in a CDI murine model and multispecies biofilms have been shown to be a reservoir for *C. difficile* spores in triple-stage chemostat human gut model (Lawley et al., 2012; Soavelomandroso et al., 2017; Normington et al., 2021). While these data clearly indicate that biofilm formation is indeed an important virulence factor our knowledge on the physiology of *C. difficile* biofilms is still scarce. Moreover, it remains to be determined whether *in vitro* biofilm model systems sufficiently resemble *in vivo* biofilm formation. Currently, *in vitro C. difficile* biofilms are often studied in plastic microtiter plates, where cells initially attach to the surface, followed by a maturation of the biofilm and a final detachment of cell-cell-aggregates (Dawson et al., 2012; Ðapa et al., 2013; Dubois et al., 2019). Similar biofilms have been observed in continuous-flow micro-fermenters (Poquet et al., 2018). Alternatively, colony biofilms on agar plates and related biofilm models have been used (Crowther et al., 2014; Semenyuk et al., 2014; James et al., 2018). Inconsistent results from *in vitro* experiments suggest that biofilm physiology strongly depends on the chosen strain, growth conditions and phase as well as the experimental setup (Ðapa et al., 2013; Maldarelli et al., 2016; Mathur et al., 2016; Pantaléon et al., 2018). For example, differences in flagella production and contribution to biofilm formation has been reported for different strains and phases of biofilm formation (Ðapa et al., 2013; Maldarelli et al., 2016). Analogously, a comprehensive RNA-seq approach revealed that the expression levels of some genes which had previously been linked to biofilm formation were significantly different between *C. difficile* colony biofilms grown on agar plates and biofilms formed on glass beads (Maldarelli et al., 2016).

Considering the strong evidence that *C. difficile* colonizes the gut in a biofilm-like manner a profound molecular characterization of *C. difficile* biofilms is crucial, e.g., for the targeted development of therapeutics and vaccines. Since most vaccines are directed against cell-surface exposed structures, the comprehensive characterization of *C. difficile’s* cell-surface proteins and polysaccharides in biofilms is of particular importance (Bruxelle et al., 2017; Kirk et al., 2017a; Péchiné et al., 2018; Bradshaw et al., 2019). Here, we applied a comparative proteomics approach to investigate the proteome repertoire of *C. difficile* strain 630Δ*erm* grown either as aggregate or colony biofilm to (i) identify proteins contributing to biofilm formation of one or the other growth condition and (ii) elucidate the underlying regulatory networks.

## Materials and Methods

### Bacterial Strains and Media

The reference strain *C. difficile* 630Δ*erm* (DSM28645) (Hussain et al., 2005; van Eijk et al., 2015) was grown at 37°C in an anaerobic workstation (Toepffer Lab Systems, Germany) in Brain Heart Infusion (BHIS; Oxoid (Thermo Fisher Scientific), Waltham, MA) supplemented with L-cysteine (0.1%(wt/vol), Sigma-Aldrich, St. Louis, MO), and yeast extract (5 mg/ml; Carl Roth, Germany) as described earlier (Ðapa et al., 2013). Prior to cultivation, spores were allowed to germinate for 72 h in BHIS medium. Subsequently, the germinated cells were used to inoculate pre-cultures which were grown for 18 h in BHIS medium. For planktonic growth main cultures were inoculated to an optical density of 0.05 at 600 nm.

### Growth of Colony and Aggregate Biofilms

The growth of colony biofilms was performed as previously described with some modifications (Semenyuk et al., 2014). Briefly, BHI medium was supplemented with yeast extract and agarose (1.5% v/v) and autoclaved for 15 min. Subsequently, sterile-filtered cysteine in BHI medium was added to the medium, mixed and transferred to petri dishes. Plates were allowed to dry for 24 h in the anaerobic chamber. Prior to cultivation, a filter membrane with a pore size of 0.45 μm (cellulose ester, Sigma-Aldrich, St. Louis, MO) was placed on top of the BHIS agar plate. Per plate 100 μl of *C. difficile*-containing BHIS medium were placed on top of the filter membrane. The BHIS medium was inoculated by an exponentially grown *C. difficile* main culture (OD_60__0n__*m*_: ∼0.5) in a 100-fold dilution. Plates were wrapped with parafilm in order to protect them from drying. The colony biofilms were grown for three and six days at 37°C in the anaerobic environment.

Aggregated biofilms were cultivated as previously reported with some modifications (Dawson et al., 2012). Briefly, sterile 6-well plates (polystyrene, Corning, NY) were filled with 2 ml *C. difficile*-containing BHIS medium. The BHIS medium was inoculated by an exponentially grown *C. difficile* main culture (OD_60__0n__*m*_: ∼0.5) in a 100-fold dilution. The 6-well plates were placed in a plastic bag to avoid evaporation of the medium. Cells were grown for three and six days at 37°C in the anaerobic environment.

### Cell Harvest and Protein Extraction

Planktonically-grown cells were harvested after 6 and 12 h of cultivation at 6.000 × g for 20 min and pellets were stored at −80°C. Filter membranes with colony biofilms were placed into 15 ml reaction tubes and stored at −80°C. In order to separate aggregated cells from free-living planktonic cells the culture-medium was carefully removed from the 6-well plates and pooled (one 6-well plate = one biological sample). The pooled culture-medium was filtered through a 10 μm filter (Isopore^TM^ PC Membrane, Merck Millipore, Tullagreen, Ireland), washed with 5 ml of 0.9% NaCl (w/v) and subsequently the filter was stored at −80°C. The filtrate containing the planktonic cells was centrifuged at 6.000 × g and the cell pellet was stored at −80°C.

The samples (cell pellets of filtrate samples and membrane filters of both biofilm types) were kept in 0.6–1 ml of an urea-containing buffer (7 M urea, 2 M thiourea, 50 mM dichlorodiphenyltrichloroethane (DDT), 4% (w/v) 3-[(3-cholamidopropyl) dimethylammonio]-1-propanesulfonate (CHAPS), 50 mM Tris-HCl). Cell lysis was performed by sonication in six cycles on ice as done previously (Sonoplus, Bandelin, Berlin, Germany, Otto et al., 2016; Berges et al., 2018). Cell debris was removed by centrifugation at 6,000 × g for 20 min at 4°C. 200 μl of the resulting lysates were precipitated by ice-cold acetone (in a 1:7 ratio v/v) for 20 h at −20°C. Subsequently, samples were allowed to warm up at RT and were centrifuged at 22,000 × g for 45 min at RT. The supernatant was discarded and the pellets were dried at RT. The protein pellets were solubilized in 100 μl of an SDS-containing urea-buffer (7 M urea, 2 M thiourea, 1% SDS v/v). In order to estimate the relative protein concentration, 10 μl of each sample was mixed with SDS-loading buffer and separated by SDS-PAGE (Criterion TGX Precast Gels 12%, Biorad, Hercules, CA, Laemmli, 1970). Protein gels were fixed for 1 h at RT (40% EtOH, 10% glacial acidic acid and 50% H_2_O), washed in H_2_O and stained by the Flamingo fluorescent dye (Biorad, Hercules, CA) for 1 h at RT. Remaining dye was removed by a washing step in H_2_O and fluorescence signals of the samples were obtained by Typhoon scanner (GE Healthcare, Little Chalfont, United Kingdom). The fluorescence signals of the gel image were quantified by ImageQuant (Biorad, Hercules, CA). These fluorescent signal intensities were used for normalization to load comparable protein amounts (30 μg of protein per sample) on the final SDS-Gel. For each sample, the entire gel lane was cut into 10 gel blocks and proteins were digested in-gel with trypsin as follows: the excised gel pieces were destained using 50% (v/v) methanol in 100 mM NH_4_HCO_3_. Subsequently, gel pieces were dehydrated using 100% ACN and allowed to dry. Modified trypsin (sequencing grade, Promega, Germany) was added to a final ratio of 1:10 (trypsin/sample) in 50 mM Tris/HCl, pH 7.5 and the sample incubated at 37°C overnight. Peptides were iteratively extracted from the gel by a four-step procedure, using ACN, 5% (v/v) formic acid in ddH_2_O and further two steps of ACN. Peptide-containing supernatants were pooled and completely dried using a Speedvac concentrator (Eppendorf AG). Samples were subsequently resolved in buffer A (5% (v/v) ACN, 0.1% (v/v) formic acid) and desalted using ZipTips (C18, Millipore). Desalted peptides were again vacuum-dried and stored at −20°C. Finally, the samples were solubilised in 10 μL 0,1% acetic acid solution and transferred into vials for MS-analysis.

### Mass Spectrometric Measurement and Data Analysis

Samples were subjected to LC-MS/MS measurements using an EASYnLC 1,000 (Thermo Fisher Scientific, Odense, Denmark) with self-packed columns [(Luna 3 μC18(2) 100A, Phenomenex, Germany)] in a one-column setup on-line coupled to an Orbitrap Elite (Thermo Fisher Scientific, Bremen, Germany) setting parameters as previously described (Berges et al., 2018).

Database search and intensity based absolute quantification (iBAQ) was achieved using the MaxQuant proteomics software package (Cox and Mann, 2008; Tyanova et al., 2016a; version: 1.6.10.43). A protein sequence database of *C. difficile* strain 630Δ*erm* containing 3781 entries was obtained from UniProt. Common laboratory contaminants and reverse sequences were added by the MaxQuant software. Parameters were set as follows: Trypsin cleavage with a maximum of two missed cleavages was assumed and oxidation of methionine was set as variable modification. Default parameters were used for protein identification. For label-free protein quantification unique and razor peptides were considered with a minimum ratio count of 2. Match between runs was enabled with default settings within each sample group. *C difficile* proteins were considered as identified if they were identified with at least two unique peptides in at least two out of three biological replicates. riBAQ values were calculated as published previously (Shin et al., 2013). Averaged riBAQs were used to calculate log2 fold changes. Proteins significantly altered in their abundance between two conditions were identified by two-way analysis of variance (ANOVA) followed by a Tukey *post hoc* test using the Perseus software package (Tyanova et al., 2016b; version: 1.6.2.2). Cellular localization of identified proteins was predicted by PSORTb (Yu et al., 2010; version: 3.0).

### Voronoi-Regulon Treemaps

Global protein expression patterns were analyzed and visualized using Voronoi treemaps (Bernhardt et al., 2013) which were adapted to illustrate regulons of several well defined global regulators of *C. difficile* as described in the literature. For this purpose, data extracted from eight transcriptomic studies was used to generate a scaffold for the Voronoi-regulon-treemaps. In these previous studies, regulons of fourteen global regulators were characterized, i.e., SigB (Kint et al., 2017), SigH (Saujet et al., 2011), SigD (El Meouche et al., 2013), SigL/RpoN (Soutourina et al., 2020), CodY (Dineen et al., 2010), CcpA (Antunes et al., 2012), Fur (Ho and Ellermeier, 2015), Hfq (Boudry et al., 2014), c-di-GMP (McKee et al., 2018b), Spo0A (Pettit et al., 2014), SigF, SigE, SigG, and SigK (Fimlaid et al., 2013). The corresponding regulons, comprising negatively as well as positively regulated genes, differ in size in the range of 30 to more than 400 genes (Table 1). In total, these transcriptional regulators modulate the expression of 1252 different *C. difficile* genes. For the illustration of the defined regulons each regulated gene was assigned to its corresponding regulator(s). Additionally, the regulatory effect (positive or negative) of a regulator on the expression of a specific gene is indicated by a plus or minus symbol. Finally, *C. difficile* log2 fold changes of biofilm models compared to filtrate samples were mapped onto the regulon maps.

**TABLE 1:** Selected regulators of *C. difficile* gene expression.

Regulator	Regulatory event	Regulon size (genes)	References
SigB	General stress response	663	Kint et al., 2017
SigH	Transition phase	490	Saujet et al., 2011
SigD	Motility, toxin production	146	El Meouche et al., 2013
SigL/RpoN	Amino acid catabolism	114	Soutourina et al., 2020
CodY	Regulation of metabolism	160	Dineen et al., 2010
CcpA	Carbohydrate catabolism	313	Antunes et al., 2012
Fur	Iron acquisition	125	Ho and Ellermeier, 2015
Hfq	Posttranscriptional (pleiotropic) regulation	203	Boudry et al., 2014
c-di-GMP	Motility	160	McKee et al., 2018b
Spo0A	Sporulation	297	Pettit et al., 2014
SigF	Sporulation (forespore)	181	Fimlaid et al., 2013
SigE	Sporulation (mother cell)	164	Fimlaid et al., 2013
SigG	Sporulation (forespore)	34	Fimlaid et al., 2013
SigK	Sporulation (mother cell)	30	Fimlaid et al., 2013

## Results and Discussion

### A Comparative Proteomics Approach to Investigate the Protein Repertoire of *C. difficile* Biofilms

**FIGURE 1:**
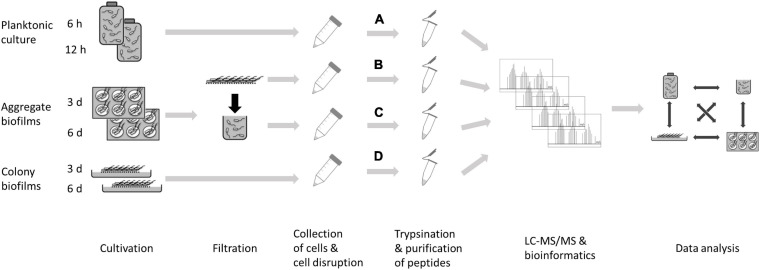
Experimental setup for a comparative proteomics approach to investigate biofilm formation in *C. difficile*. *C. difficile* strain 630Δerm was grown as planktonic culture for 6 and 12 h **(A)**, or as aggregate biofilm in six-well plates for 3 or 6 days **(B)** or as colony biofilm on solid growth medium for 3 or 6 days **(D)**. In addition, cells from the filtrate of aggregate biofilms were analyzed **(C)**. Cells were harvested and their protein repertoire was subsequently analyzed using a LC-MS/MS approach.

In order to identify proteins required for and/or characteristic of *C. difficile* biofilm formation the protein inventory of biofilm- and planktonically-grown bacteria was comparatively analyzed. Two different types of biofilms were investigated: 1. colony biofilms on agar plates and 2. aggregate biofilms formed in 6-well plates. Although colonies on agar plates do not meet the traditional criteria of a biofilm, the densely packed cells likewise present a form of multicellular growth attached to a biotic surface (Gingichashvili et al., 2017; Kesel et al., 2017) and it cannot be excluded that the growth conditions that bacteria face during this form of multicellular living are relevant during infection. Colony biofilms were directly analyzed after scraping them off the plates. Aggregate biofilms from 6-well plates were harvested by filtration and cells that passed the filter were analyzed in parallel as non-biofilm fraction. Previous studies investigating *C. difficile* biofilm gene expression by RNA-seq used planktonically-grown cells as reference (Maldarelli et al., 2016; Poquet et al., 2018). Thus, planktonically-grown cells from exponential (6 h) and stationary (12 h) growth phase were also included in this study (Figure 1).

Principal component analyses revealed that the various proteome parameters obtained for every tested condition clustered together within the biological replicates but revealed a clear cut separated distribution in dependence of the growth condition and time (Supplementary Figure 1). This was also true for the data obtained for cells from the filtrate (sample C of Supplementary Figure 1) of the aggregate biofilms which clearly separated from the corresponding biofilm cells (sample B of Supplementary Figure 1), although both types of cells experienced the same cultivation time and growth medium along with nutrient limitation and accumulated waste products. We propose physiological differences between these cells to be mainly attributed to cell aggregation/biofilm formation and therefore used the filtrate samples as the reference for non-biofilm conditions rather than the planktonic cells (sample A of Supplementary Figure 1) which were not further considered.

**FIGURE 2:**
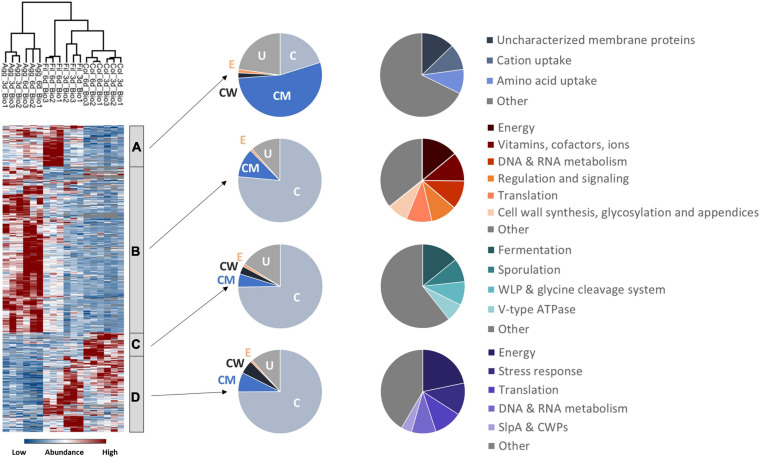
Hierarchical cluster analyses of *C. difficile* proteome data sets. The heatmap on the left side shows differentially abundant proteins between aggregate biofilms (Agg), filtrate cells (Fil), and colony biofilms (Col) from day three (3d) and day six (6d) for three biological replicates (Bio1-3). Z-transformed abundance values are displayed as a color gradient where blue colors indicate weakest abundance levels and red colors the strongest abundance levels among the growth conditions with maximum z-scores of +/–1.5. Hierarchical clustering revealed four major cluster **(A–D)** representing 6-day old filtrate cells cluster **(A)**, aggregate biofilms **(B)**, colony biofilms **(C)** or both, colony biofilms and filtrate samples **(D)**. In the pie charts on the right the various clusters shown on left were further analyzed with regard to the subcellular location (middle: C for cytoplasmic; CM for cytoplasmic membrane; CW for cell wall; E for extracellular; U for unassigned). In addition, proteins within a cluster were grouped according to their function and the most prominent functional groups of each cluster are presented in the pie charts on the right (WLP for Wood-Ljungdahl pathway). A detailed list of all significantly differentially abundant proteins including fold changes can be found in Supplementary Table 2 (Supplementary Material). Agg: Aggregate biofilms; Col: Colony biofilms; Fil: Filtrate samples; 3d: Samples from day three; 6d: Samples from day six; C, cytoplasmic; CM, cytoplasmic membrane; CW, cell wall; E, extracellular; U, unassigned; WLP, Wood-Ljungdahl pathway; CWPs, cell wall proteins.

Global hierarchical cluster analyses on all proteins that were found differentially expressed between the six remaining data sets using ANOVA (analysis of variance) yielded four major clusters A–D (Figure 2 and Supplementary Table 2). Cluster A contained proteins with higher abundance in 6-day old filtrate cells and consisted of a high proportion of membrane proteins (54%) while all other clusters contained approximately 75% cytosolic proteins. In line with this, cluster A contained transporters for cations and amino acids. The largest cluster B contained 607 proteins of high abundance in aggregate biofilms which were assigned to the functional categories “cell wall biosynthesis”, “cell surface polysaccharide biosynthesis”, “flagella biosynthesis”, “regulation and cell signaling”, and “amino acid and carbohydrate metabolism”. Cluster C was composed of 99 proteins highly abundant in colony biofilms, and the 267 proteins of cluster D were found in higher abundance in colony biofilms and filtrate cells. Both clusters share a high proportion of cell-wall proteins including the S-layer protein SlpA and 12 of the 28 cell wall proteins (CWPs) encoded in one operon with SlpA. Additionally, “fermentation and energy generation” proteins of cluster C were assigned to the Wood-Ljungdahl pathway and the corresponding glycine cleavage system, and to subunits of the V-type ATPase, but also “sporulation”-related proteins such as CotA, CotB, and SipL were observed.

In agreement with transcriptome data comparing biofilm and non-biofilm cells (Maldarelli et al., 2016; Poquet et al., 2018), we identified genes encoding cell-surface exposed proteins, but also genes from the categories “energy metabolism”, “stress response”, “virulence” and “regulation and cell signaling” as biofilm signature.

### Production of Cell Surface-Associated Proteins in *C. difficile* Biofilms

**FIGURE 3:**
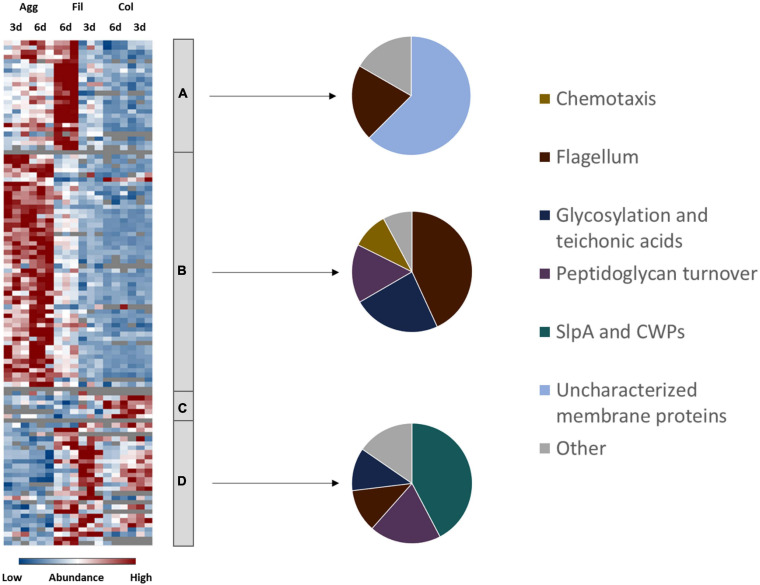
Differential abundance of cell surface-associated proteins. 108 surface-associated proteins were found differentially abundant (blue is down, red is up) between aggregate biofilms (Agg), filtrate samples (Fil) and colony (Col) from day three (3d) and day six (6d). Z-transformed abundance values are displayed as a color gradient where blue colors indicate weakest abundance levels and red colors the strongest abundance levels among the growth conditions with maximum z-scores of +/–1.5. The heatmap on the left site shows that they were found in all 4 clusters, representing the 6-day old filtrate cells cluster **(A)**, aggregate biofilms **(B)**, colony biofilms **(C)** or both, colony biofilms and filtrate samples **(D)**. Pie charts on the right reveal that each individual cluster comprised mostly proteins from a specific functional group of cell surface-associated proteins. A list of the underlying proteins for this heatmap including their fold changes in abundance can be found in Supplementary Table 3. Agg, Aggregate biofilms; Col, Colony biofilms; Fil, Filtrate samples; 3d, Samples from day three; 6d, Samples from day six; CWPs, cell wall proteins.

Adhesion to epithelial cells as well as cell-cell-aggregation is mainly mediated by cell-surface exposed proteins, polysaccharides and cell appendices such as pili and flagella (Ðapa et al., 2013; McKee et al., 2018a; Arato et al., 2019). Notably, proteins from the respective categories were found differently expressed between colony and aggregate biofilms (Figure 3 and Supplementary Table 3). Most flagella proteins (i.e., FliE, FliF, FliG, FliH, FliM, FlgE, FlgG, FhlA, FhlF) and type IV pili proteins (i.e., PilT, PilB2, and PilM2) were detected in higher concentrations in aggregate biofilms than in colony biofilms and filtrates. Similar behavior was observed for proteins involved in cell wall biogenesis like enzymes of the *mur* operon (i.e., MurB, MurE, MurG), enzymes involved in lipoteichoic acid synthesis (GtaB, GtaB1) and modification (DltA), enzymes of the Cell Wall Glycopolymer (CWG) locus (CD2783 to CD2769), and proteins of membrane-lipid biosynthesis encoded by the *fab* operon (i.e., FabD, FabH, FabK). In contrast, the S-layer protein SlpA and most CWPs (i.e., Cwp12, Cwp16, Cwp19, Cwp22, Cwp84) were of significantly lower abundance in aggregate biofilms.

#### Flagella and Type IV Pili

While comparable RNA-seq based expression profiles were observed for cell wall and S-layer biogenesis genes when aggregate biofilms and planktonic cells were compared (Poquet et al., 2018), transcription of flagella genes has been found to be reduced in aggregate and colony biofilms in comparison to planktonic cells of *C. difficile* (Maldarelli et al., 2016; Poquet et al., 2018). However, both transcriptomic studies were performed with biofilms grown in different setups and to different timepoints. Hence, flagella might be required for aggregate biofilm growth in our rather static set up, but might be obsolete or even obstructive for biofilm formation in continuous flow systems (Poquet et al., 2018) or for biofilms grown on glass beads (Maldarelli et al., 2016). As discussed above, somewhat contradictory observations have been obtained regarding the role of flagella during *C. difficile* biofilm formation (Ðapa et al., 2013; Jain et al., 2017) that may either reflect the complexity of the regulatory system underlying flagella expression in *C. difficile* (El Meouche et al., 2013; Soutourina et al., 2013; Anjuwon-Foster and Tamayo, 2017, 2018) and/or the different functions of flagella that depend on their posttranslational modification state (Twine et al., 2009; Faulds-Pain et al., 2014; Bouché et al., 2016; Valiente et al., 2016). Additionally, biofilms are often composed of subpopulations (Vlamakis et al., 2008; Besharova et al., 2016) and flagella in one or the other modification state might be relevant for one subpopulation and obstructive for the other. Alterations of flagella post-translational modifications were shown to impact motility and cell-cell-aggregation properties of *C. difficile* (Faulds-Pain et al., 2014; Valiente et al., 2016). Significant upregulation of *C. difficile’s* conserved glycosyltransferase CDIF630erm_00362 in conjunction with the flagella proteins in aggregate biofilms in our study might suggest that flagella in aggregate biofilms were glycosylated. The CDIF630erm_00362 gene is encoded directly downstream of *fliC* which is essential for flagella formation and the bacterium’s virulence *in vivo* (Valiente et al., 2016). Obviously, flagella are not always required for biofilm formation in *C. difficile* but might be beneficial depending on their state of modification in some cases.

In contrast, there is growing consensus that type IV pili are dispensable for early biofilm development, but required in mature biofilms of *C. difficile* (Maldarelli et al., 2016; Purcell et al., 2016; McKee et al., 2018a). We found type IV pili-associated proteins like PilT, PilB2 and PilM2 in aggregate biofilms and in lower amounts in filtrates but not in colony biofilms. Therefore, our data suggest that aggregate biofilms in contrast to colony biofilms obviously utilized glycosylated flagella and certain pili components during their formation.

#### Cell Surface Glyco-Polymers and Teichoic Acids

Proteins from the PSII locus for the synthesis of the teichoic-acid-like cell-surface polysaccharide II (e.g., CDIF630erm_03033, CDIF630erm_03035, CDIF630erm_03041), GtaB and GtaB1 providing UDP-glucose for teichoic acid synthesis and DltA involved in D-alanylation of teichoic acids were detected in aggregate biofilms and in lower amounts in the filtrates, and even lower or not in colony biofilms. Polysaccharide II is one of three of *C. difficile’s* cell surface polysaccharides (Ganeshapillai et al., 2008; Reid et al., 2012; Willing et al., 2015). The ubiquitously found polysaccharide, attached to the cell surface, represents either an important antigen or is masking surface antigens with higher inflammatory potential. Its presence in *C. difficile* biofilms was reported before (Ðapa et al., 2013; Chu et al., 2016). Likewise, lipoteichoic acids revealed a significant immunogenic potential. They have been found to fulfill numerous functions in antibiotic resistance, cell wall homeostasis, cell division and metabolism (McBride and Sonenshein, 2011; Schade and Weidenmaier, 2016).

#### The S-Layer

The S-layer covers the cell surface of *C. difficile* and consists of the main S-layer protein SlpA which is encoded in one operon with 28 CWPs. *C. difficile’s* S-layer protein SlpA and the 28 CWPs have been shown to be immunogenic (Péchiné et al., 2011; Bruxelle et al., 2016; Mizrahi et al., 2018). Deletion of the S-layer encoding genes renders a pathogenic *C. difficile* strain apathogenic (La Riva et al., 2011; Kirk et al., 2017b; Vaz et al., 2019). Furthermore, the S-layer, was found to play a role in cell-cell-aggregation and attachment to epithelial cells (Waligora et al., 2001; Merrigan et al., 2013; Bradshaw et al., 2017; Kirk et al., 2017b; Richards et al., 2018). Interestingly, the proteome data revealed that SlpA and 23 of its adjacent CWPs could be identified in colony biofilms and filtrate samples but were almost not detected in aggregate biofilms. This is in line with observations made by Janoir et al. (2013) who reported the downregulation of SlpA in the late stage of a mouse colonization model. The cysteine protease Cwp084, which was among the lower abundant CWPs, is responsible for the proteolytic processing of S-layer proteins (Kirby et al., 2009; Gooyit and Janda, 2016). Interestingly, a *cwp84* mutant produces more biofilm mass compared to the wildtype (Pantaléon et al., 2015). Since the mutant was unable to cleave the SlpA precursor protein, a different more hydrophobic surface and different matrix proteome composition was observed, which might lead to an enhanced surface attachment of the cells, potentially explaining the increased biofilm production (La Riva et al., 2011; Pantaléon et al., 2015). Pantaléon et al. (2015) further detected most CWPs in the biofilm matrix (Cwp5, Cwp6, Cwp 9, Cwp14, Cwp21) and supernatant (CwpV, Cwp2, Cwp11, Cwp12, Cwp13, Cwp16, Cwp18, Cwp19, Cwp22, Cwp25, Cwp66) rather than in the surface proteome by comparative MALDI TOF analyses.

#### The Potential Role of PrkC Kinase During Biofilm Formation of *C. difficile*

Cwp7 was one of the few CWPs that revealed a differential expression pattern between the analyzed growth conditions and was significantly lower abundant in colony biofilms than in filtrates although not as low in aggregate biofilms as most other CWPs. Interestingly, a previous investigation showed that deletion of the membrane-associated serine/threonine kinase PrkC which is involved in cell wall homeostasis and antimicrobial resistance led to the downregulation of almost all CWPs but to an upregulation of Cwp7. Additionally, the Δ*prkC* mutant showed an increased release of polysaccharide II into the supernatant, was less motile and produced more biofilm (Cuenot et al., 2019). Although PrkC was found to be significantly higher abundant in the 3-day old aggregate biofilms and significantly lower abundant in 6-day old colony biofilms, the similar expression pattern of aggregate biofilms and the Δ*prkC* mutant suggest a possible role of PrkC in the regulation of biofilm formation in *C. difficile*.

In summary, aggregate biofilms analyzed here were characterized by higher abundance of potentially glycosylated flagella, type IV pili proteins, proteins required for cell surface polysaccharide synthesis and proteins involved in cell wall and membrane turnover compared to colony biofilms while SlpA and CWPs were lower abundant in aggregate biofilms compared to colony biofilms.

### Energy Metabolism

The unique energy metabolism of *C. difficile* preferentially utilizes amino acids through a process called Stickland fermentation which firstly produces ATP via substrate level phosphorylation (Stickland, 1934; Neumann-Schaal et al., 2015). Secondly, various carbohydrates are the basis for a complex mixed acid fermentation (Riedel et al., 2017; Hofmann et al., 2021). Some of the involved processes are coupled via the membrane associated Rnf complex to the formation of an ion gradient which in turn drives ATP formation via a classical F_*O*_F_1_-ATPase (Müller et al., 2008). Alternative carbon sources like ethanolamine are used additionally (Nawrocki et al., 2018; Hofmann et al., 2021). When preferred amino acids and glucose are depleted from the medium, *C. difficile* is able to generate energy from lactate fermentation and to fix CO_2_ via the Wood-Ljungdahl pathway (Köpke et al., 2013; Hofmann et al., 2021).

**FIGURE 4:**
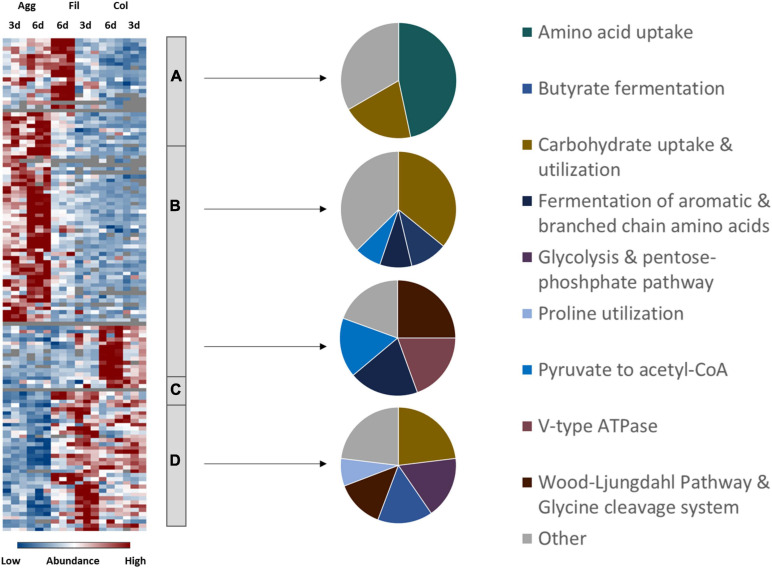
Differential abundance of energy metabolism-associated proteins. 185 metabolism-related proteins were found differentially abundant between aggregate biofilms (Agg), filtrate samples (Fil) and colony (Col) from day three (3d) and day six (6d). Z-transformed abundance values are displayed as a color gradient where blue colors indicate weakest abundance levels and red colors the strongest abundance levels among the growth conditions with maximum z-scores of +/–1.5. The heatmap on the left site shows that they were found in all 4 clusters, representing the 6-day old filtrate cells cluster **(A)**, aggregate biofilms **(B)**, colony biofilms **(C)** or both, colony biofilms and filtrate samples **(D)**. Pie charts on the right reveal that each individual cluster comprised mostly proteins from a specific functional metabolic pathway. A list of proteins from this heatmap including their fold changes in abundance can be found in Supplementary Table 4. Agg, Aggregate biofilms; Col, Colony biofilms; Fil, Filtrate samples; 3d, Samples from day three; 6d, Samples from day six.

Although all biofilm and filtrate cells were obviously subject to nutrient limitations due to extended incubation time, proteomic profiles for proteins involved in various pathways of energy generation indicated an aggregate- and colony biofilm specific energy metabolism (Figure 4 and Supplementary Table 4).

#### Amino Acid Utilization

Several systems of the oxidative and reductive branches of the Stickland amino acid fermentation were found differentially produced under the different biofilm conditions investigated in comparison to the filtrate samples. To start with, proteins from the *CDIF630erm_00522-…-etfA1* operon (AcdB, EtfA1, EtfB1) for the reductive fermentation of leucine and phenylalanine to 3-phenylproprionate/isocoproate and the activator protein of the 2-hydroxyisocaproyl-CoA dehydratase, HadI, were higher abundant in aggregate biofilms compared to other culture conditions. The reductive fermentation of leucine and phenylalanine is indirectly coupled to the Rnf complex via ferredoxin (Kim et al., 2006; Schiffels and Selmer, 2019). In agreement, proteins from the Rnf complex (RnfB, RnfC, RnfD, RnfG) were higher abundant in filtrate and aggregate biofilms than in colony biofilms. In contrast, the proline reductase (PrdA, PrdB) for reductive degradation of proline to 5-aminovalerate, which is directly coupled to the Rnf complex (Jackson et al., 2006), showed the highest abundance levels in filtrate samples. Similarly, the subunits of the glycine reductase (GrdB, GrdC, GrdD) and associated proteins TrxA2 and TrxB3 for degradation of glycine to acetate were higher abundant in filtrate cells than in biofilm cells. Enzymes of the oxidative branch for fermentation of branched chain (VorA1, VorB1, VorC1, Ptb1) and aromatic amino acids (CDIF630erm_02622, IorA, IorB, Ptb1) were higher abundant in colony biofilms. To react to the reduced availability of nutrients, both filtrate samples and aggregate biofilms were found to have transporters for the uptake of amino acids such as those encoded by the *app* operon for oligo-peptide transport and MetNQ required for methionine uptake. In contrast, colony biofilms showed an overall lower abundance of any kind of transporters which might reflect the even more impaired diffusion of nutrients inside the dense biofilm matrix. It cannot be excluded that membrane protein extraction was less efficient for colony biofilms and reinforced this observation. However, overall only a very minor bias toward cytoplasmic and against membrane proteins was observed and significant changes in protein formation between the different analyzed conditions caused by regulatory processes were clearly visible.

#### Carbohydrate Utilization

*C. difficile* degrades carbohydrates via glycolysis and the pentose-phosphate pathway yielding pyruvate (Hofmann et al., 2018). Proteome analysis showed that enzymes of both pathways, such as PfkA, Pgi, Tpi, and Eno from the glycolysis and Tal, Tal1, RpiB1, RpiB2, Rpe1, and Tkt’ from the pentose-phosphate pathway, were higher abundant in filtrate samples. This is in contradiction to results of Poquet et al. (2018) who reported induction of glycolysis and pentose-phosphate pathway in aggregate biofilms compared to planktonically-grown cells. However, both data sets agree on active carbohydrate utilization within aggregate biofilms. As observed for amino acid uptake systems, both filtrate samples and aggregate biofilms revealed the presence of PTS systems for the uptake of carbohydrates. Moreover, aggregate biofilms revealed an extensive amount of enzymes for the utilization of carbohydrates which underlines the importance of carbohydrates for *C. difficile* under infection-relevant conditions (Theriot et al., 2014; Jenior et al., 2017, 2018; Fletcher et al., 2018). Also, Dubois et al. (2019) reported that induction of biofilm formation by deoxycholate was enhanced in the presence of fermentable carbohydrates.

#### Ethanolamine Utilization

An abundant nutrient source in the gut is the membrane lipid phosphatidylethanolamine. The derived amino alcohol ethanolamine can serve as carbon and nitrogen source. Availability of ethanolamine has been shown to reduce virulence in *C. difficile* and to delay the onset of CDI in a hamster model (Nawrocki et al., 2018). Interestingly, proteins involved in ethanolamine utilization were more or less exclusively identified in filtrate cells. However, e.g., in *Entercoccus faecalis* ethanolamine utilization is dependent on the presence of cobalamin (Del Papa and Perego, 2008). Assuming similar regulation in *C. difficile*, induction of cobalamin biosynthesis pathways in the aggregate biofilms (Supplementary Table 1) suggests that these biofilms were limited in cobalamin what might explain the inhibition of ethanolamine utilization gene expression under these growth conditions.

#### Utilization of the Intermediate Products Pyruvate and Acetyl-CoA

The intermediate product of the glycolysis as well as alanine oxidation, pyruvate, is further metabolized to either acetyl-CoA and formate via the pyruvate formate-lyase PflDE or to lactate by the lactate dehydrogenase Ldh (Dannheim et al., 2017; Hofmann et al., 2018). Acetyl-CoA is in turn degraded to acetate by Ptb1 and AckA or to butyrate via the butyrate fermentation pathway encoded in the *bcd2-…-thlA* and *4hbD-…-CDIFerm_02583* operons (Hofmann et al., 2018). Formate can be further metabolized to hydrogen by the formate hydrogenases FdhD and FhdF and the hydrogenases HydN1, HydN2, and HydA (Berges et al., 2018). According to our proteome data, several proteins for butyrate fermentation (ThlA1, Hbd, Crt2, EtfB3, Ptb, AbfD) and the hydrogenase HydN2 were found in higher amounts in colony biofilms and in lower amounts in aggregate biofilms. In contrast, the electron bifurcating lactate dehydrogenase encoded by CDIF630erm_01319-01321 was detected in higher amounts in aggregate biofilms. Pyruvate formate-lyase PflDE was found induced in biofilms compared to filtrate cells.

#### Expression of F_*O*_F_1_-Type and V-Type ATPase

Rarely observed among bacteria, *C. difficile* encodes for two ATPases. In addition to its F_*O*_F_1_-ATPase, which uses the membrane potential to generate ATP, *C. difficile* encodes a Na ^+^–or H ^+^ -transporting V-type ATPase which uses ATP to maintain the ion gradient across the membrane and which has been linked to the Wood-Ljungdahl pathway and glycine cleavage system before (Saujet et al., 2011; Poquet et al., 2018). While the subunits of the F_*O*_F_1_-ATPase were overall lower abundant in both biofilm types compared to filtrate cells, the V-type ATPase subunits were significantly higher abundant in colony biofilms but less abundant in aggregate biofilms compared to filtrate cells. This suggests that the V-type ATPase is of importance for the colony biofilms.

#### Role of the Wood-Ljungdahl Pathway

In line with this, several proteins from the Wood-Ljungdahl pathway (WLP) were higher abundant in colony biofilms while repressed in aggregate biofilms in comparison to filtrate cells. With the exception of AcsABE that were found higher abundant in aggregate biofilms, almost all enzymes of the WLP, such as the carbon monoxide dehydrogenase (CooS, CDIFerm_00297, CDIFerm_00298), Fhs, FchA, FoldD, MetV and MetF, and the glycine cleavage system proteins (GcvTPA, GcvPB), which provide 5,10-methylene-tetrahydrofolate for the WLP, were significantly higher abundant in colony biofilms but lower abundant in aggregate biofilms compared to filtrate cells. The WLP, also known as the reductive Acetyl-CoA pathway, is able to generate acetyl-CoA from CO_2_ (Stupperich et al., 1983; Köpke et al., 2013). Moreover, the WLP has recently been suggested as an electron sink to maintain cell homeostasis in the absence of Stickland acceptors (Gencic and Grahame, 2020). Taken together, the higher levels of proteins from the oxidative branch of Stickland fermentation, of butyrate fermentation and of the V-type ATPase discussed above as well of WLP proteins in colony biofilms strongly support the hypothesis that the WLP plays an important role in maintaining cell growth in environments depleted in reductive equivalents and potentially maintains the membrane potential in concert with the coupled V-type ATPase (Gencic and Grahame, 2020). Of note, Poquet et al. (2018) reported a downregulation of WLP, glycine cleavage system and V-type ATPase genes in aggregate biofilms compared to planktonic cells which is in line with results of this proteome analysis.

In conclusion, higher production of proteins for less favorable energy pathways such as the Wood-Ljungdahl pathway and the lactate dehydrogenase and lower production of proteins for more favorable energy pathways and enzymes such as the proline and glycine reductase as well as glycolysis in biofilm cells compared to filtrate cells suggest that biofilms were more affected by the nutrient limitation than filtrate cells. However, both biofilms obviously responded differently. Presence of several PTS and enzymes for carbohydrate uptake and degradation and the induction of cofactor biosynthesis proteins in aggregate biofilms suggest that aggregate biofilms are (1) rather active cells that are able to invest ATP to take up nutrients from the environments and (2) permeable enough to allow nutrients to reach cells inside the biofilm. In contrast, the observed uniform induction of WLP, V-type ATPase, butyrate fermentation and oxidative branch of the Stickland fermentation in colony biofilms compared to aggregate biofilm and filtrate cells likely points at severe limitation of reductive equivalents in colony biofilms that rather supports survival than reproduction.

### Stress Response and Virulence

**FIGURE 5:**
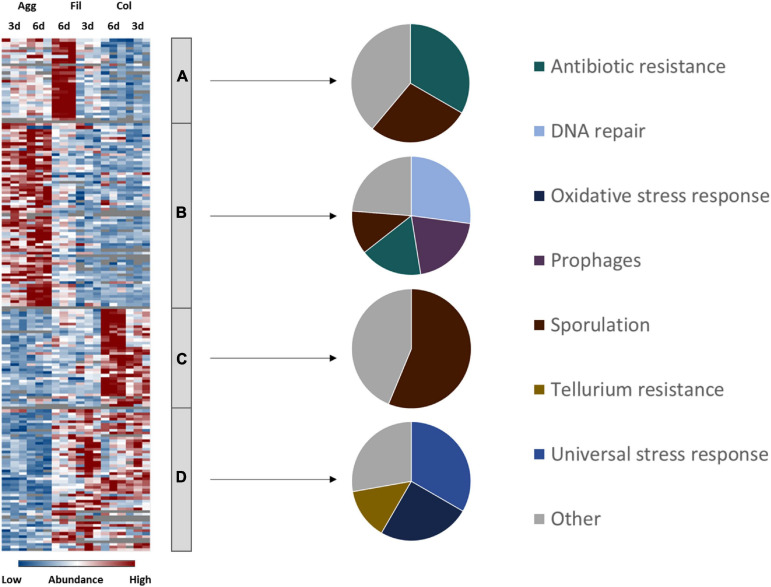
Differential abundance of virulence, antibiotic resistance and stress response-associated proteins. 129 proteins assigned to virulence, antibiotic resistance and stress-response related pathways were found differentially abundant (blue is down, red is up) between aggregate biofilms (Agg), filtrate samples (Fil) and colony (Col) from day three (3d) and day six (6d). Z-transformed abundance values are displayed as a color gradient where blue colors indicate weakest abundance levels and red colors the strongest abundance levels among the growth conditions with maximum z-scores of +/–1.5. The heatmap on the left site shows that they were found in all 4 clusters, representing the 6-day old filtrate cells cluster **(A)**, aggregate biofilms **(B)**, colony biofilms **(C)** or both, colony biofilms and filtrate samples **(D)**. Pie charts on the right reveal that each individual cluster comprised proteins from specific stress response and resistance systems. A list of proteins from this heatmap including their fold changes in abundance can be found in Supplementary Table 5 (Supplementary Material). Agg, Aggregate biofilms; Col, Colony biofilms; Fil, Filtrate samples; 3d, Samples from day three; 6d, Samples from day six.

In general, biofilm cells are embedded inside an extracellular matrix that protects cells from antibiotics and disinfectants but at the same time also impairs diffusion of nutrients and waste products (Anwar et al., 1992; Karygianni et al., 2020). Consequently, cells from the inner biofilm have to cope with nutrient limitation and accumulation of waste products. To survive such stressful conditions *C. difficile’s* genome encodes for various stress response systems as a first line of defense (Sebaihia et al., 2006). If conditions remain unfavorable, *C. difficile* initiates toxin synthesis or, as a last resort, sporulation (Onderdonk et al., 1979; Lawley et al., 2009; Underwood et al., 2009). Analysis of *C. difficile’s* stress reponse systems revealed that both biofilm types and filtrate cells revealed different expression of stress response and virulence-associated pathways similar to the results for the energy metabolism (Figure 5 and Supplementary Table 5).

#### Stress Response

As a strictly anaerobic pathogen, *C. difficile* requires an effective oxidative stress response to be able to react to oxygen and reactive oxygen species (Neumann-Schaal et al., 2018). Interestingly, our proteome data set revealed that some of *C. difficile’s* oxidative stress response proteins, such as the rubrerythrin Rbr and the reverse rubrerythrins Rbr2 and Rbr3, were drastically lower abundant in aggregate biofilms compared to filtrate samples but slightly higher abundant in colony biofilms than in filtrate samples. Since PerR, a transcriptional regulator which represses oxidative stress response proteins, is inactive in strain Δerm due to a single nucleotide polymorphism in the *perR* gene (Troitzsch et al., 2021), these effects are possibly a result of post-transcriptional regulation. On the other hand, 6-day old colony biofilms revealed an induction of some oxidative stress response proteins such as NorR and SodA. Since no molecular oxygen was present in any of the tested conditions, the oxidative stress response proteins identified here were either expressed to react to other oxidative species such as reactive nitrogen species or other yet unknown signals not present in aggregate biofilms. Similarly, other stress response-associated proteins such as DnaK, GrpE, GroL, and ClpC, which were previously shown to be induced in response to heat stress, bile acids and antibiotics (Jain et al., 2011; Ternan et al., 2012; Chong et al., 2014; Sievers et al., 2019), were significantly lower abundant in aggregate biofilms compared to filtrate samples while the transcriptional regulators CtsR and HrcA, which repress the above mentioned proteins in other firmicutes species (Schulz and Schumann, 1996; Derré et al., 1999), were higher abundant in aggregate biofilms compared to the filtrate samples. In general, aggregate biofilms seemed to face less stress than colony biofilms and filtrate samples.

#### Antibiotic Resistance

In contrast, antibiotic resistance-associated proteins such as ClnA and ClnR involved in cationic antimicrobial peptide resistance (Woods et al., 2018), the tetracycline resistance protein TetM (Mullany et al., 1990) and putative multidrug ATP-type transport proteins such as CDIF630erm_00291, CDIF630erm_00940, and CDIF630erm_02245 revealed highest protein levels in aggregate biofilms, but were rarely detected in colony biofilms. Indeed, most studies addressing antibiotic resistance of *C. difficile* biofilms consistently showed that biofilms are more resistant to various antibiotics such as metronidazole (Semenyuk et al., 2014), vancomycin and linezolide (Tijerina-Rodríguez et al., 2019). In addition to impaired diffusion through the dense extracellular matrix and differential regulation of antibiotic resistance markers (Høiby et al., 2010) increased mutation rates as a result of accumulating metabolic waste products as well as increased horizontal gene transfer that is often observed in biofilms where cell densities are particularly high are assumed to boost antibiotic resistance of biofilms (Molin and Tolker-Nielsen, 2003; Boles and Singh, 2008; Levin and Cornejo, 2009; Ryder et al., 2012). Accordingly, levels of proteins required for homologous recombination and DNA repair (RecN, RuvB, RadA, UvrABC, MutLS, SbcCD, LexA) were significantly higher in aggregate biofilms than in filtrate samples. In line with this, it was shown before, that the induction of the SOS response (RecA, UvrABC) in a *lexA* deletion mutant resulted in an increased biofilm mass further demonstrating the importance of gene transfer and genetic evolution for efficient biofilm formation (Walter et al., 2015). Again, colony biofilms revealed even lower protein amounts of mentioned proteins than filtrate cells.

#### Toxin Synthesis

Both forms of biofilms had in common a decreased production of toxin A and B compared to filtrate samples although the effect was more pronounced in aggregate biofilms. In agreement, toxin B mRNA levels were previously determined to be lower in aggregate biofilms than in colony biofilms. Toxin A mRNA levels were found to be decreased in aggregate biofilms vs. planktonic cells (Maldarelli et al., 2016; Poquet et al., 2018). Worth mentioning, toxin expression in *C. difficile* underlies a sophisticated regulatory network that is tightly coupled to the energy metabolism (Dineen et al., 2007; Antunes et al., 2011; Dubois et al., 2016; Hofmann et al., 2018). In summary, although both biofilms revealed significantly different expression profiles with regard to energy metabolism, toxins were found downregulated in both biofilm models indicating that the biofilm lifestyle rather facilitates persistence than infection.

#### Sporulation

Although biofilms were initially assumed to be hot spots of sporulation and a potential reservoir for spores during persistence, recent data suggest that this may not be the case and only a few spores can be found in *C. difficile* biofilms which additionally were determined to be different from other spores with regard to germination efficiency and heat resistance (Ðapa et al., 2013; Semenyuk et al., 2014; Pizarro-Guajardo et al., 2016; Dubois et al., 2019). Moreover, it was reported that sporulation rates in biofilms vary between strains and do not correlate with severity of disease (Semenyuk et al., 2014). In agreement with the previous observations, we determined that sporulation and spore proteins such as spore coat proteins CotA, CotB, SipL, and SpoIVA were less abundant in aggregate biofilms than in filtrate samples which matches the concomitant higher abundance of the negative regulators of sporulation, KipI and Soj, in aggregate biofilms (Ðapa et al., 2013; Poquet et al., 2018; Dubois et al., 2019). In contrast, we found spore proteins significantly higher abundant in colony biofilms. This, however, matches the observation that the carbohydrate utilization- and the amino acid uptake systems App and Opp, whose expression was found negatively correlated with sporulation before (Antunes et al., 2012; Edwards et al., 2014), as well as proteins involved in translation, ribosome maturation and cell division such as RumA, MiaB, BipA, Obg, and InfB showed lower abundance in our colony biofilms than in aggregate biofilms and filtrate samples. Again, these data indicate a higher metabolic activity of *C. difficile* in aggregate biofilms and in filtrate samples compared to colony biofilms.

Taken together, the lower response levels of aggregate biofilms to unknown metabolic stresses and lower sporulation rates but higher production of antibiotic resistance markers suggest that the extracellular matrix of aggregate biofilms is possibly less dense than in colony biofilms what insufficiently protects cells from antibiotics but prevents accumulation of waste products and allows diffusion of nutrients. While Poquet et al. (2018) who cultivated aggregate biofilms in continuous flow systems argued that the constant renewal of medium is responsible for the observed metabolic activity and low sporulation rates, the data presented here indicate that aggregate biofilms comprise active growing cells regardless of the nutrient supply. Overall, the differential expression of sporulation proteins depending on the choice of biofilm model is an interesting observation and further underlines the urgent need to answer the question of which type of biofilm is produced in the host.

### Global Regulatory Circuits and Cell Signaling

**FIGURE 6:**
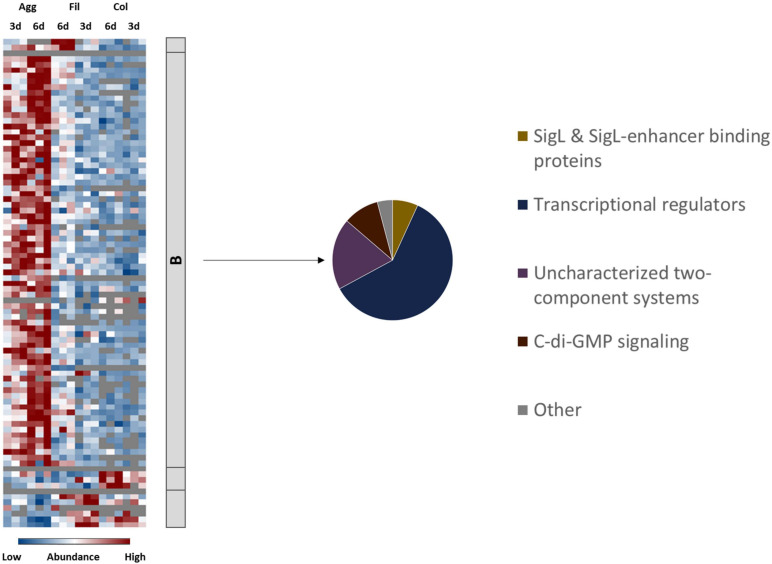
Differential abundance of signaling and regulatory proteins. 84 proteins annotated as regulatory or signaling proteins were differentially abundant (blue is down, red is up) between aggregate biofilms (Agg), filtrate samples (Fil) and colony (Col) from day three (3d) and day six (6d). Z-transformed abundance values are displayed as a color gradient where blue colors indicate weakest abundance levels and red colors the strongest abundance levels among the growth conditions with maximum z-scores of +/–1.5. The heatmap on the left site shows that they were found in all 4 clusters, representing the 6-day old filtrate cells cluster **(A)**, aggregate biofilms **(B)**, colony biofilms **(C)** or both, colony biofilms and filtrate samples **(D)**. The pie chart on the right reveals that proteins from Cluster B comprised proteins from four major groups of regulatory and signaling proteins. A list of proteins from this heatmap including their fold changes in abundance can be found in Supplementary Table 6 (Supplementary Material). Agg, Aggregate biofilms; Col, Colony biofilms; Fil, Filtrate samples; 3d, Samples from day three; 6d, Samples from day six.

Finally, proteins involved in regulation and cell signaling were analyzed to uncover which regulatory circuits possibly underlie the observations discussed in the previous sections. In view of the extensive remodeling of the cell envelope and of metabolic pathways and the tight control of toxin synthesis and sporulation, it was not surprising that multiple central regulatory networks have been affected during biofilm formation in *C. difficile* (Ðapa et al., 2013; Purcell et al., 2017; Dubois et al., 2019). For example, c-di-GMP signaling and quorum sensing were shown to be required for biofilm formation before (Ðapa et al., 2013; Purcell et al., 2017; Slater et al., 2019). Moreover, several publications have reported that deletion of various regulatory proteins including Spo0A (Dawson et al., 2012; Ðapa et al., 2013), Hfq (Boudry et al., 2014), CcpA (Dubois et al., 2019), CodY (Dubois et al., 2019), and as mentioned above LexA (Bordeleau et al., 20119) impaired biofilm formation in *C. difficile*. In agreement, proteome data sets presented here suggested that especially aggregate biofilm formation involved complex gene regulatory network restructuring and induction of various transcriptional regulators and two component system proteins (Figure 6 and Supplementary Table 6).

**FIGURE 7:**
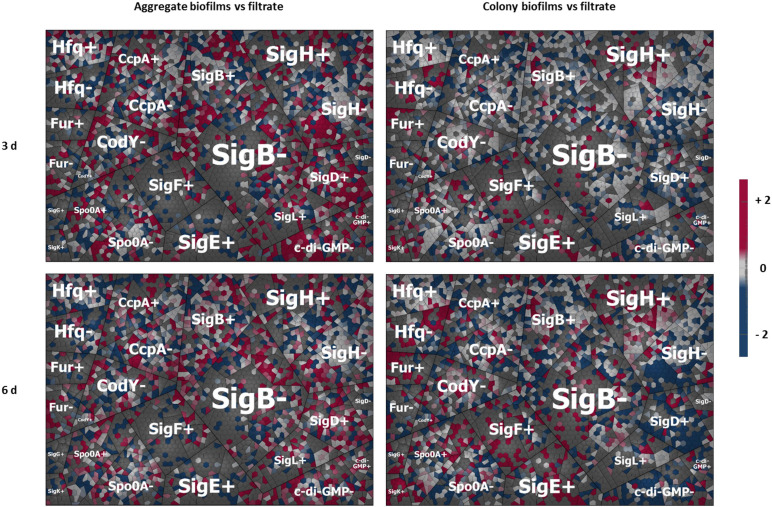
Regulon maps for aggregate and colony biofilms both vs. filtrate samples. Shown are four Voronoi regulon treemap maps. They are summarizing the mapping of our proteome data onto *C. difficile’s* hitherto characterized regulons created based on available gene expression (transcriptome) data from the literature. All proteins regulated by a respective regulator were depicted as single cells clustered together with all other protein under the control of the same regulator either positively (+) or negatively (–) controlled by the respective regulators. The log2 fold changes of each biofilm sample relative to the corresponding filtrate sample were directly mapped to visualize the impact of a respective regulator on the sample set. Red cells represent proteins induced in biofilm samples relative to filtrate samples while blue cells present proteins downregulated in biofilms relative to filtrate samples. The upper left panel shows the data for aggregate biofilm vs. the filtrate sample after 3 days (3d); lower left panel aggregate biofilms after 6 days (6d); upper right panel colony biofilm after 3 days; lower right panel colony biofilms after 6 days. “+” stands for proteins positively regulated by the respective regulator, “–” for proteins negatively regulated by the respective regulator. A detailed list of all proteins including fold changes and additional information on regulon and operon affiliation can be found in Supplementary Table 6.

In order to visualize and analyze the complex gene regulatory networks that were active in the different biofilm setups and to potentially assign some of the afore-discussed features of each biofilm type to specific regulatory systems, regulon analyses were performed. To do so, available global expression data of *C. difficile* strains deleted or overexpressed in central regulators were used to assign *C. difficile* proteins to their respective regulons. Subsequently, our proteome data were mapped onto obtained regulons and the results were visualized in Voronoi treemaps (Figure 7).

#### Key Regulators of Colony Biofilms

In accordance with the observed induction of sporulation proteins in colony biofilms discussed above, the obtained regulon maps revealed the induction of regulons for the sporulation sigma factors SigE, SigF, SigG, and SigK and the master regulator of sporulation, Spo0A, in the 6-day old colony biofilms (Figure 7). While SigE and SigF were previously shown to be not required for biofilm formation (Dubois et al., 2019), Spo0A was frequently reported to be essential for biofilm formation in *C. difficile* (Ðapa et al., 2013; Dubois et al., 2019). Moreover, proteins from the SigH and Fur regulon were found upregulated in colony biofilms. Upregulation of Fur proteins might be the result of the impaired diffusion of ions inside the biofilm matrix. The sigma factor SigH is required during transition from exponential growth to stationary phase and was demonstrated to regulate sporulation, toxin production and surface-associated proteins (Saujet et al., 2011). Several of the proteins and operons found to be differentially abundant between colony and aggregate biofilms, such as the flagella operons, the S-layer protein SlpA, the V-type ATPase operon, the oxidative stress response and the glycine reductase complex, were shown to be under control of SigH suggesting that SigH might considerably shape the colony biofilm metabolism (Saujet et al., 2011). In contrast, proteins from the regulon of the motility regulator SigD and the c-di-GMP regulon were rather low abundant in colony biofilms.

#### Activation of c-di-GMP Signaling in Aggregate Biofilms

c-di-GMP signaling relies on the production of the second messenger c-di-GMP. In contrast to other Gram-positive bacteria, *C. difficile’s* genome encodes for a large number of functional diguanylate cyclases and phosphodiesterases suggesting special importance of c-di-GMP signaling in *C. difficile* (Bordeleau et al., 2011). Out of these c-di-GMP associated proteins, the diguanylate cyclases CDIF630erm_01581, CDIF630erm_02043, and CDIF630erm_03665, and the phosphodiesterases PdcA, CDIF630erm_00875 and CDIF630erm_01792 were found significantly upregulated in aggregate biofilms compared to filtrate samples or were even exclusively identified in aggregate biofilms. As shown previously, elevated c-di-GMP levels promote expression of type IV pili that subsequently mediate attachment of the gut bacteria to epithelial cells, cell aggregation and biofilm formation in *C. difficile* strain R20291 (Purcell et al., 2012, 2016; McKee et al., 2018a). In contrast, flagella proteins that are known to be negatively regulated by c-di-GMP were highly abundant in the aggregate biofilm samples indicating that other regulatory circuits likely interfere with c-di-GMP signaling (Purcell et al., 2012; McKee et al., 2013, 2018b).

#### The Role of SigL/RpoN in Aggregate Biofilms

Our data suggest that the alternative sigma factor SigL/RpoN, encoded by CD630_31760 and also known as σ^54^, plays an important role in the gene regulation of aggregate biofilms (Figures 6, 7). The SigL/RpoN regulon has frequently been observed to significantly contribute to biofilm formation in various species by positive regulation of motility (Jagannathan et al., 2001; Saldías et al., 2008; Francke et al., 2011; Iyer and Hancock, 2012), enhancement of extracellular DNA levels inside the extracellular matrix (Iyer and Hancock, 2012) and remodeling of metabolic pathways to adapt to nutrient conditions inside the biofilm (Arous et al., 2004; Francke et al., 2011; Xu et al., 2014; Hayrapetyan et al., 2015). Initially described as sigma regulator induced by nitrogen limitation (Hirschman et al., 1985; Weiss and Magasanik, 1988) SigL/RpoN was later found in most bacterial species to be activated in response to various stressful conditions, such as osmostress and low temperatures (Francke et al., 2011; Nie et al., 2019). The SigL/RpoN regulon in *C. difficile* and other *Clostridiales* has recently been characterized leading to the discovery of 30 SigL/RpoN-dependent promoters. Of these 30 SigL/RpoN-dependent promoters, 23 were adjacent to a gene sequence encoding for a SigL/RpoN-dependent regulator, a group of regulators also known as enhancer binding proteins (EBP). Thereby SigL/RpoN was identified as an important central regulator controlling amino acid and carbohydrate catabolism pathways in *C. difficile* (Nie et al., 2019; Soutourina et al., 2020). Interestingly, 15 of the 23 proposed EBPs for *C. difficile* were identified in this study. Thirteen EBPs were solely present or detected in higher amounts in aggregate biofilms compared to filtrate samples and colony biofilms suggesting an important role of the SigL/RpoN regulon during aggregate biofilm formation. Indeed, the activation of the SigL/RpoN regulon most likely explains some of the energy-related observations discussed above. For instance, the *CDIF630erm_00522-…-etfA1* operon for reductive fermentation of branched chain amino acids, which was higher abundant in aggregate biofilms compared to filtrate samples, is positively controlled by the EBP LeuR which was among the EBPs higher abundant in aggregate biofilms. Similarly, several PTS systems are under control of SigL/RpoN and its EBPs (Soutourina et al., 2020). For instance, the galactitol-specific PTS component CDIF630erm_00104, the mannose-specific PTS components CDIF630erm_00408 and CDIF630_00409 and the ribitol-specific PTS component CDIF630erm_00620 were either found in higher amounts or exclusively in aggregate biofilms. Since toxin synthesis was recently found to be negatively controlled by SigL/RpoN in *C. difficile*, also the low production of toxins in aggregate biofilms might be a result of SigL/RpoN regulation (Dubois et al., 2016). Overall, our proteome data suggest that SigL/RpoN has an important role in aggregate biofilm formation.

#### Other Regulatory Circuits Involved in Aggregate Biofilm Formation

Furthermore, the regulon maps revealed a slightly higher induction of proteins from the SigB, CcpA, and CodY regulons in aggregate biofilms than in colony biofilms which may have contributed to the remodeling of the energy metabolism in aggregate biofilm cells (Dineen et al., 2010; Antunes et al., 2012; Kint et al., 2017). In addition, activation of SigB may have contributed to repression of sporulation proteins (Kint et al., 2017). Minor changes in the Spo0A regulon in aggregate biofilms suggest that Spo0A is possibly required for initial or early biofilm formation or cellular adaptation in general rather than maturation and maintenance of biofilm homeostasis. In contrast, the quorum sensing protein LuxS, which was previously shown to be essential for biofilm formation in *C. difficile* (Ðapa et al., 2013), was decreased in both aggregate and colony biofilms compared to filtrate cells. LuxS was shown to contribute to biofilm formation via induction of prophage genes that in turn induce cell lysis resulting in higher levels of eDNA in the biofilm matrix of strain R20291 (Slater et al., 2019). Possibly, LuxS is more important in early biofilm formation due to its activation in late exponential phase (Carter et al., 2005) or rather required for adaptation to pre-longed survival in nutrient depleted conditions which is a pre-requisite of biofilm formation. Of note, proteome data of this study still demonstrated induction of prophage genes in aggregate biofilms despite the low abundance of LuxS (Figure 5 and Supplementary Table 5).

To conclude, the prominent synthesis of SigL/RpoN, some of its EBPs and SigL/RpoN-dependent operons such as the operon for branched chain amino acid utilization in aggregate biofilms suggest that SigL is an important regulator of aggregate biofilm formation. In addition, c-di-GMP likely shaped the protein inventory of aggregate biofilms while colony biofilms were most likely shaped by the sporulation sigma factors Spo0A, SigE, SigF, SigG, and SigK as well as sigma factor SigH and the transcriptional regulator Fur. However, it should be stressed that biofilm formation is obviously a dynamic process that involves successive action of various regulatory proteins and pathways. Thus, the data of the presented two sample points only allow a temporal narrow view. Moreover, regulon data were not available for all regulators so that the impact of these regulators might be missed. Furthermore, proteins are often under control of several regulators what makes it difficult to unequivocally link adaptation processes to a certain regulator. Nevertheless, the data provide novel in-depth insight into the complex regulatory network of biofilm formation in *C. difficile* and likely permit helpful conclusions of the necessity of specific regulators which are less prone to bias than regulator knock-out studies.

## Conclusion

The comprehensive proteome data presented here shed light on the protein repertoire of *C. difficile* strain 630Δ*erm* grown in two different biofilm models. Based on the results, we confirmed that free-floating aggregate biofilms behave drastically different from sessile colony biofilms and could possibly be the more relevant biofilm model. Cells of this biofilm type were characterized by significant metabolic activity, flagellation and activation of various regulatory circuits while they neither produced spores or toxins (Table 2), which is in line with previous transcriptomic data (Maldarelli et al., 2016; Poquet et al., 2018). Three aspects might explain some discrepancies observed between previously reported transcriptome approaches, e.g., by Poquet et al. (2018), and the here presented proteome data set. First, discrepancies could be explained by the initially discussed different experimental set ups used to grow biofilms (6-well plates vs. continuous flow system). Second, the choice of the reference sample sets applied (filtrate cells of same age as biofilms vs. 24 h batch cultures) impedes direct comparison of data sets. Lastly, previous observations demonstrated that transcriptome and proteome data do not necessarily follow the same expression trend (Zapalska-Sozoniuk et al., 2019). Therefore, we would like to emphasize that, although detection of a protein should not be mistaken for the protein’s activity, proteome data probably reveal a more reliable picture of what genes are active within the two biofilm models than transcriptome data could do and might subsequently be more relevant for vaccine and antimicrobial design. On that basis, type IV pili were validated as important cell-surface antigens in *C. difficile* biofilms while the role of flagella and the S-layer remains unclear (Table 2). Moreover, we did not only confirm the importance of c-di-GMP signaling in biofilm formation but further suggested a central role for SigL/RpoN in *C. difficile* biofilms and conclude that research on this well-conserved regulator is hitherto underrepresented (Table 2). Of note, Tremblay et al. (2021) recently suggested a role for SigL/RpoN in *C. difficile* biofilms. Likewise, the data presented here emphasize the importance to characterize various still uncharacterized transcriptional regulators and two-component systems of which several were induced in aggregate biofilms (Table 2). Generally, further research is necessary to completely decipher the process of biofilm formation in *C. difficile* and to identify the nature of infection-relevant biofilms. Thereby, comprehensive *in vivo* omics studies including proteomic based studies will be inevitable. Moreover, integration of such *in vivo* data, which to some extent already exist for animal model studies and analyses of patients’ stool samples, with *in vitro* data such as those presented in this study will be just as important. For instance, transcriptomic and metabolomic data obtained from mice experiments suggest that *C. difficile* consumes significant amounts of amino acids but also of several carbohydrates such as mannitol during colonization of the intestine (Theriot et al., 2014; Jenior et al., 2017; Pereira et al., 2020). Consequently, the discussed identification of carbohydrate uptake and utilization pathways in the aggregate biofilms might be highly relevant for infection conditions.

**TABLE 2:** Differential protein abundance between aggregate and colony biofilms of C. difficile.

Category	Pathway/function	Aggregate biofilms	Colony biofilms
Cell surface proteins	Flagella	**↑**	**↓**
	Cell surface glycosylation	**↑**	**→**
	S-layer	**↓**	**→**
Energy metabolism	V-type ATPase	**↓**	**↑**
	Wood-Ljungdahl-Pathway	**↑**	**↑**
	Stickland fermentation–ox. Branch	**↓**	**↑**
	Stickland fermentation–red. Branch	**↑**	**↓**
	Glycolysis	**↓**	**↓**
	Pentose phosphate pathway	**↓**	**↓**
Stress response	Antibiotic resistance	**↑**	**↓**
	DNA repair	**↑**	**↓**
	Ox. Stress response	**↓**	**→**
	Chaperones	**↓**	**→**
	Toxins	**↓**	**↓**
	Sporulation	**↓**	**↑**
	c-di-GMP signaling	**↑**	**↓**
			
Regulation and signaling	Quorum sensing	**↓**	**↓**
	Two-component systems	**↑**	**↓**
	SigL/RpoN regulon	**↑**	**↓**

*The protein inventory of C. difficile aggregate and colony biofilms was analyzed revealing differences mainly in the categories “Cell surface proteins,” “Energy metabolism,” “Stress response” and “Regulation and cell signaling.” Pathways or proteins with respective functions were either less abundant (↓), higher abundant (↑) or remained unchanged (→).*

## Data Availability Statement

The datasets presented in this study can be found in online repositories. The names of the repository/repositories and accession number(s) can be found below: https://www.ebi.ac.uk/pride/archive/, PXD022830.

## Author Contributions

CL, KR, and DJ designed the research. CL and CH performed the research. DB and JH were involved in mass spectrometric analyses. MB and CL analyzed the data. MB, CL, DJ, SS, and KR conceptualized and wrote the manuscript. All authors contributed to the article and approved the submitted version.

## Conflict of Interest

The authors declare that the research was conducted in the absence of any commercial or financial relationships that could be construed as a potential conflict of interest.
