# Supplementary figures and images for: What’s a Biofilm?—How the Choice of the Biofilm Model Impacts the Protein Inventory of Clostridioides difficile
Source: Front Microbiol. 2021 Jun 10;12:682111. doi: 10.3389/fmicb.2021.682111 (PMC8225356; doi:10.3389/fmicb.2021.682111)

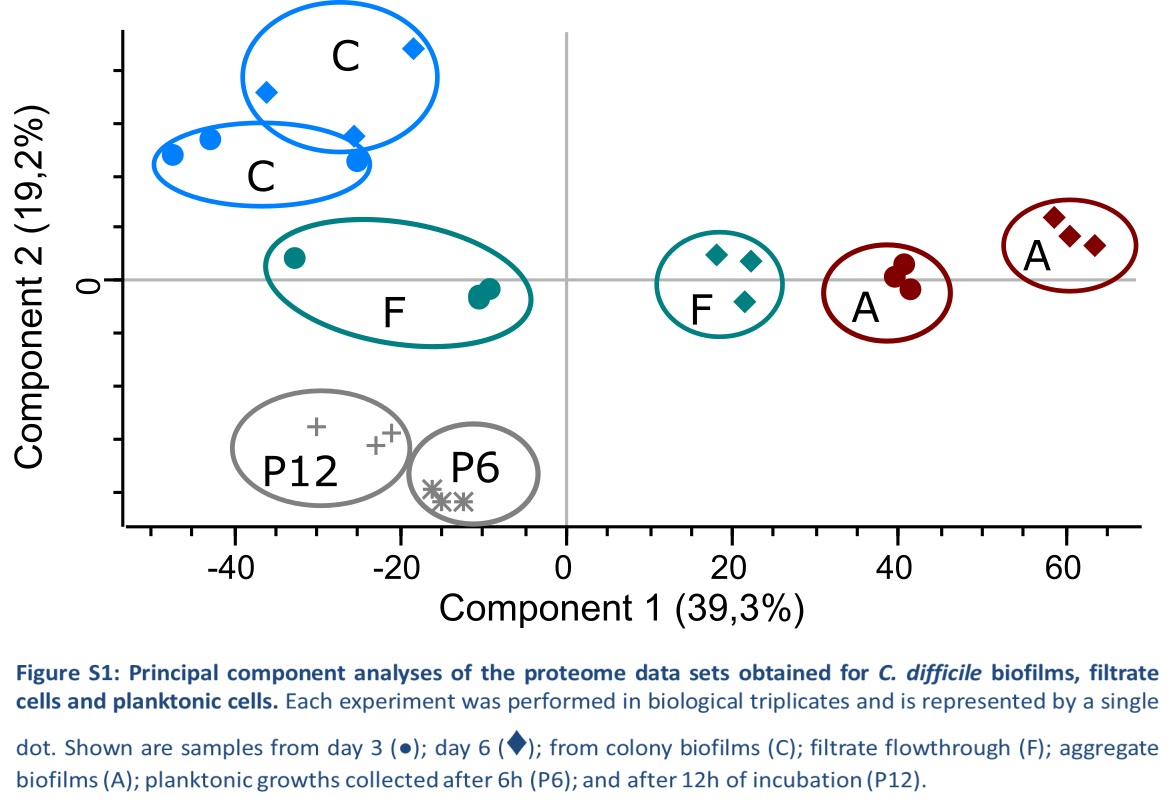

Supplement: Supplementary file 2 [file Image_1.JPEG]
